# Case report: a giant arachnoid cyst masking Alzheimer’s disease

**DOI:** 10.1186/s12888-019-2247-8

**Published:** 2019-09-05

**Authors:** Anna-Sophia Wahl, Martin Löffler, Lucrezia Hausner, Michaela Ruttorf, Frauke Nees, Lutz Frölich

**Affiliations:** 1 Department of Geriatric Psychiatry, Central Institute of Mental Health, Medical Faculty Mannheim, Heidelberg University, J5, 68159 Mannheim, Germany; 2 Department of Cognitive and Clinical Neuroscience, Central Institute of Mental Health, Medical Faculty Mannheim, Heidelberg University, Mannheim, Germany; 3 Computer Assisted Clinical Medicine, Medical Faculty Mannheim, Heidelberg University, Mannheim, Germany

**Keywords:** Arachnoid cysts, Cognitive decline, Alzheimer’s disease, Functional neuro-imaging, Neural plasticity

## Abstract

**Background:**

Intracranial arachnoid cysts are usually benign congenital findings of neuroimaging modalities, sometimes however, leading to focal neurological and psychiatric comorbidities. Whether primarily clinically silent cysts may become causally involved in cognitive decline in old age is neither well examined nor understood.

**Case presentation:**

A 66-year old caucasian man presenting with a giant left-hemispheric frontotemporal cyst without progression of size, presented with slowly progressive cognitive decline. Neuropsychological assessment revealed an amnestic mild cognitive impairment (MCI) without further neurological or psychiatric symptoms. The patient showed mild medio-temporal lobe atrophy on structural MRI. Diffusion tensor and functional magnetic resonance imaging depicted a rather sustained function of the strongly suppressed left hemisphere. Amyloid-PET imaging was positive for increased amyloid burden and he was homozygous for the APOEε3-gene. A diagnosis of MCI due to Alzheimer’s disease was given and a co-morbidity with a silent arachnoid cyst was assumed. To investigate, if a potentially reduced CSF flow due to the giant arachnoid cyst contributed to the early manifestation of AD, we reviewed 15 case series of subjects with frontotemporal arachnoid cysts and cognitive decline. However, no increased manifestation of neurodegenerative disorders was reported.

**Conclusions:**

With this case report, we illustrate the necessity of a systematic work-up for neurodegenerative disorders in patients with arachnoid cysts and emerging cognitive decline. We finally propose a modus operandi for the stratification and management of patients with arachnoid cysts potentially susceptive for cognitive dysfunction.

**Electronic supplementary material:**

The online version of this article (10.1186/s12888-019-2247-8) contains supplementary material, which is available to authorized users.

## Background

Intracranial arachnoid cysts are usually benign congenital lesions, filled with cerebrospinal fluid and covered by arachnoidal cells and collagen. They typically develop between the surface of the brain and the cranial base or on the arachnoid membrane. Widespread usage of non-invasive neuroimaging modalities have made the diagnosis of arachnoid cysts one of the most common incidental findings: While in a healthy population slightly less than 3% of subjects were found to have intracranial abnormalities [1], the prevalence of arachnoid cysts has been estimated to be between 0.3 and 1.7% of the general population and highest in the pediatric subpopulation [2–5]. Most intracranial arachnoid cysts remain clinically silent so that patients may live their entire life without symptoms, even if cysts are large. For symptomatic cysts, when patients present with signs of normal pressure hydrocephalus (gait disturbance, incontinence and dementia) and speech impairment, surgical cyst decompression may lead to an improvement [6, 7].

We here present the case of a 66-year-old man with a large left-hemispheric cyst and mild cognitive impairment. Although at first glance a cognitive decline seemed to be related to the anatomic anomaly, further extensive evaluation using different imaging modalities and biomarkers was required to reveal Alzheimer’s disease rather than the arachnoid cyst as underlying cause for the cognitive deterioration. With this case report we not only aim at presenting the coincidental finding of a large cyst and a neurodegenerative disease where the neurodegenerative component might have been overlooked without adequate diagnostic tools. We also discuss that arachnoid cysts mask and may further augment neurodegenerative processes. We thus suggest a modus operandi for the diagnosis and treatment of patients with arachnoid cysts and suspected cognitive impairment.

## Case presentation

### Methods

#### Patient

A 66-year-old man with a previously diagnosed giant arachnoid cyst presented at the memory clinic of the Department of Geriatric Psychiatry of the Central Institute for Mental Health Mannheim, University of Heidelberg, Germany, for further assessment and therapeutic counseling of mild, gradual short-term memory deficits.

#### Clinical, EEG and laboratory assessments

Clinical information were obtained by personal interviews and detailed medical records by trained psychiatrists. All previous clinical data were reviewed at the Central Institute of Mental Health, Mannheim. For neuropsychological assessment, the Consortium to Establish a Registry for Alzheimer’s Disease (CERAD) test battery, the Mini-Mental State Examination (MMSE), the Wechsler Memory Scale logical memory (WMS-LM) and the Verbal Learning and Memory Test (VLMT), the Clock-Drawing Test and the Rey Complex Figure Test were performed. For the CERAD and WMS, raw data were transformed in specific values and converted in z-scores adapted for sex, age and education for comparison to the general population. We interviewed the patient’s spouse based on the Alzheimer’s Disease Cooperative Study - Activity of Daily Living (ADCS-ADL) checklist. The Edinburgh Handedness Inventory was used to assess the patient’s dominance for handedness in everyday activities (Table 1). A routine standard EEG recording using the 10–20 system was performed and analyzed visually. A detailed laboratory screening was performed to exclude secondary dementias according to the current guideline of the German Societies of Neurology and Psychiatry (DGN/DGPPN S3-guideline revision1, http://www.awmf.org/uploads/tx_szleitlinien/038-013l_S3-Demenzen-2016-07.pdf).

**Table 1:** Edinburgh Handedness Inventory depicting the left-handedness of the patient. However, the patient was trained to write with his right hand at school

Edinburgh Handedness Inventury		
	Left	Right
Writing		xx
Drawing		xx
Tossing		xx
Using scissors	xx	
Toothbrush	xx	
Knife	xx	
Spoon		xx
Broom-leading hand	x	
To light a match	xx	
Open a box	x	
Dominant foot for kicking s.th.		x
Usage of the dominant eye	x	

‘x’ depicts movements mainly performed with the respective hand, ‘xx’ depicts movements that are solely performed with the respective hand

#### Neuroimaging

##### Sensorimotor task

The patient performed visually cued voluntary movements of individual digits in the scanner. The movement performed was a button press (=flex and extend back) on an optical response keypad (LUMItouch) with five response buttons, with the respective digit: digit 1 (D1: thumb), digit 2 (D2: index finger), digit 3 (D3: middle finger), digit 4 (D4: ring finger), and digit 5 (D5: little finger). Movements were performed after a visual signal. For each digit the patient received visual cues in the form of a hand drawing with the respective finger being colored. We used a block-design with movement blocks of 12 s and rest blocks of 10, 11, 12, 13 or 14 s (randomized) duration. The duration of the rest blocks was varied to avoid synchronization of stimulation and fMRI signals. During movement blocks, a visual cue instructed the patient to perform movements of a specific digit at 1 Hz (e.g., D2, D2, D2, D2.. .). Each movement block was separated by a rest block. Six movement blocks per digit were acquired (30 movement blocks in total). One block of all 5 digits was performed before any block was repeated. This was done to avoid timing effects on the BOLD signal. The order of blocks within one of these repetitions was randomized. In total the task consisted of 30 movement blocks (5 digits, 6 repetitions) and 31 rest blocks (1 in the beginning, 1 in the end of the experiment, 29 between movement blocks), summing up to a total of 12 min and 12 s, which equals 488 acquired EPI volumes (see below). This task was performed twice, once with the digits of the left and once with the digits of the right hand.

#### Magnetic resonance imaging (MRI) data acquisition

##### Diagnostic MRI

The magnetic resonance images for diagnosis were acquired on a 1 T MAGNETOM Harmony whole-body scanner (Siemens Healthineers, Erlangen, Germany) using a standard head coil. For diagnostic purposes, a sagittal T_2_ turbo spin echo (TSE) sequence (repetition time (TR) = 3420 ms, echo time (TE) = 91 ms, field of view (FoV) = 240 × 240 mm^2^, flip angle (α) = 170°, bandwidth (BW) = 150 Hz/px) was used. Nineteen slices were acquired (voxel size = 0.5 × 0.5 × 6.0 mm^3^). A coronal 2D T_1_-weigthed spin echo (SE) sequence (TR = 485 ms, TE = 10 ms, FoV = 230 × 201 mm^2^, α = 90°, BW = 100 Hz/px) was used. Twenty-three slices were acquired (voxel size = 0.5 × 0.5 × 6.0 mm^3^). Axial fluid attenuated inversion recovery (FLAIR) images were acquired using the following parameters: TR = 8000 ms, TE = 124 ms, inversion time = 2500 ms, FoV = 230 × 201 mm^2^, α = 150°, BW = 180 Hz/px. Twenty-four slices were acquired (voxel size = 0.5 × 0.5 × 6.0 mm^3^).

All diagnostic MR images were evaluated visually by an experienced neuroradiologist, medio-temporal lobe atrophy was measured by the semi-quantitative MTA-rating scale (Scheltens scale), the amount of white matter degeneration was rated using the semi-quantitative Fazekas scale [8].

##### Structural and functional MRI (fMRI)

All structural and functional magnetic resonance images were acquired on a 3 T MAGNETOM Trio whole-body scanner (Siemens Healthineers, Erlangen, Germany) using a 32-channel head coil. All T_1_-weighted images were obtained using a high-resolution magnetization prepared rapid gradient echo (3D MPRAGE) sequence (TR = 1900 ms, TE = 2.72 ms, FoV = 250 × 250 mm^2^, α = 9°, BW = 200 Hz/px). Two hundred and twenty-four axially-oriented slices were acquired (voxel size = 0.8 × 0.8 × 0.8 mm^3^). Parallel acceleration technique (iPAT) with generalized auto-calibrating partially parallel acquisition (GRAPPA) reconstruction was used with an acceleration factor of 2. Before all measurements, shimming of the scanner was done to account for maximum magnetic field homogeneity. For acquisition of fMRI data, a gradient-echo echo-planar (EPI) T_2_* sensitive sequence (TR = 1500 ms, TE = 22 ms, FoV = 220 × 220 mm^2^, α = 90°, BW = 1262 Hz/px) was used. Twenty-two AC/PC aligned slices (voxel size = 1.8 × 1.8 × 1.8 mm^3^, no gap) were acquired in interleaved slice order using GRAPPA acceleration factor 3. For acquisition of diffusion weighted images, a spin-echo EPI (TR = 8400 ms, TE = 84 ms, FoV = 192 × 192 mm^2^, BW = 1930 Hz/px) was used. Seventy slices (voxel size = 2.0 × 2.0 × 2.0 mm^3^, no gap) were acquired in interleaved slice order using GRAPPA acceleration factor 2. Diffusion weighting was performed in multi-directional diffusion weighting (MDDW) mode along 64 non-collinear directions with b = 1000 s/mm^2^. Additionally, a single non-diffusion weighted volume (b = 0 s/mm^2^) was acquired. For image registration purposes, a single-volume high-saturation EPI whole brain image was acquired with the same parameters and slice placement as in the task EPI protocol described above but with different TR (4410 ms) and containing more slices (80).

##### Amyloid-positron imaging tomography (A-PET) imaging

A- PET scanning was performed using Florbetaben ([18F]FBB). The amyloid imaging was acquired with a Siemens ECAT-47 PET tomograph. The [18F] FBB PET data (47 slices; slice thickness 3.3 mm) was summed for 90 min p.i. followed by a static 3-dimensional emission-scan over 15 min. The acquired PET data underwent standard reconstruction and images were visually assessed for amyloid positivity by a specialist trained in nuclear medicine. An established binary (amyloid-positive versus amyloid negative) scoring system was used [9, 10].

#### MRI data processing and analysis

For our analyses, we used Freesurfer software [11]. First, using the structural T1-weighted image, we generated a model of the cortical surface —involving intensity normalization, skull removal, segmentation, and tessellation. The segmentation was edited using control point procedures in freesurfer (semi-automated editing), which were followed up by manual editing.

Second, for fMRI statistical map analyses, time-series data were first motion corrected in three spatial dimensions to reduce the effects of small head motion [12]. To improve image registration the EPI images from the fMRI task were registered to the whole brain EPI image before registration to the high resolution anatomical image. Surface smoothing of 5 mm FWHM was applied and all the analyses were conducted within individual subject space. Activation in sub-regions was deemed significant at a threshold of *p* < 0.001. Cortical parcellation was performed with an automatic labelling of the cortical structures based on the Desikan-Killiany anatomical atlas. For results of cortical parcellation for pre- and postcentral gyrus see Additional file 1: Figure S1 and as illustration of the segmentation results see Additional file 2: Figure S2 and Additional file 3: Figure S3. For DTI analyses, we used FMRIB software library 5.0.10 [13]. After eddy correction and rotation of b vectors, we used dtifit routine with weighted least-squares regression to calculate fractional anisotropy (FA) and eigenvector maps.

#### Review of literature

For a systematic review of literature we searched PubMed with the key words ‘arachnoid cyst’ and ‘cognitive impairment’/‘cognitive decline’/‘dementia’. This revealed a total of 44 references. We excluded studies in children (9/44), papers with no detailed information of cyst location and/or symptoms of the patients (14/44), other comorbidities (3/44) or without an English abstract available (4/44). We included 14 clinical studies and case reports (Table 3), which presented subjects with frontotemporal arachnoid cysts and cognitive decline or other neurological symptoms. We thus evaluated the cases of 47 patients for symptomatic presentation, diagnosis and treatment.

## Results

The patient presented at the memory clinic for the assessment of slowly progressive short-term memory deficits over the course of the past 3 years. He reported troubles in finding words, forgetting names of friends and items he intended to buy in the supermarket. He described a normal developmental history with no developmental delay or psychosocial abnormalities. He remembered that he was teased by other children because of his large head and that even as a small child he would have required headgears in adult size. He denied any history of head trauma, toxic exposure, chronic headaches or another neurological or psychiatric illness. The patient had never used any psychiatric drug before. There was no history of learning disorders, hyperactivity or significant academic difficulties as well as no form of substance abuse. He completed high school and professional training as a bank accountant, where he continued to work for 27 years followed by several years of work in a communication company. The patient has stable and good social relationships. He is retired but still assisting in a pharmacy by delivering medicine to clients.

At the time of examination, his spouse confirmed cognitive decline over several years, and reported a tendency of participating less in conversations, while otherwise his psychosocial functioning was normal. She also had noticed an impaired orientation in new surroundings and episodes of confusion in group discussions or family meetings.

**Fig. 1:**
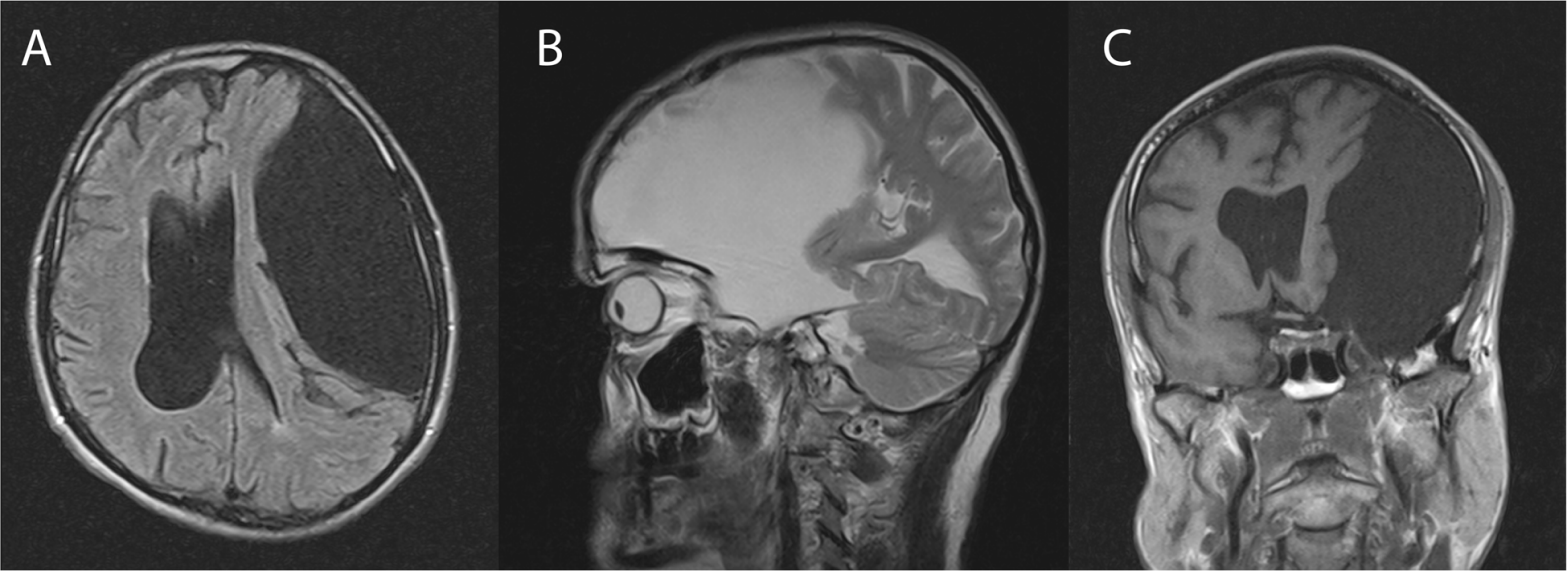
Brain MRI showing a large left frontotemporal cerebrospinal fluid-filled cyst with a cross-sectional area of 6 × 14 cm compatible with a Type III arachnoid cyst after the Galassi classification of middle cranial fossa arachnoid cysts: **a** FLAIR axial, **b** T2 TSE sagittal, and **c** T1 SE coronal view

Three years ago he had consulted a neurologist for his memory impairment, who initiated a structural cranial MRI. This revealed a large left frontotemporal cerebrospinal fluid-filled cyst with a cross-sectional area of 6 × 14 cm compatible with a Type III arachnoid cyst according to Galassi classification [14] of middle cranial fossa arachnoid cysts (Fig. 1). Two subsequent MRIs within the following 3 years did not show any size progression. On repeated neurosurgical evaluation it was decided for watchful waiting and against surgical intervention because of the absence of focal neurological signs and cyst size progression.

### Neurological and psychiatric examination

Psychiatric examination was unremarkable except for mild cognitive dysfunction. At the time of evaluation, the patient’s mood was reported stable and euthymic (5 out of 60 points in the Montgomery-Asberg Depression Rating Scale).

The neurological examination was normal. There were no clinical signs of hydrocephalus (no speech and gait problems, incontinence or papilledema) detected. Although the patient was mainly left-handed (Edinburgh Handedness Inventory, Table 1) he was trained to write with his right hand. However, he kept preferentially using his left hand for all fine skilled motor function and sports (tennis, squash). We also performed a detailed laboratory screening according to the current guideline of the German Societies of Neurology and Psychiatry (see methods) and could exclude hints for secondary forms of dementia.

### Electroencephalography (EEG) recordings

EEG showed a distinct 10 Hz alpha rhythm with normal topographical distribution and no lateral or focal abnormalities and no signs of increased cerebral excitability. The visual blockade reaction was normal. There were no signs of increased slow wave activity.

### Neuropsychological assessment

On neuropsychological assessment no significant deficits were detectable in the CERAD test battery for most of the tests. However his performance in discriminability was mildly impaired (Table 2). The patient’s overall level of cognitive function, phonetic fluency, figure savings and constructional praxis recall were on the lower border range compared to a sex-, age- and education- adapted representative sample set of the general population. With the WMS we aimed to assess the patient’s mnestic performance for complex verbal learning matter. While the patient completed the immediate recall task on average level, his assessment was below average in the delayed recall after 30 min (<− 1 standard deviation, Table 2, B). We also tested the patient in the VLMT, which evaluates the learning performance, consolidation of long-term memory and the recognition of a list consisting of 15 words. Although the patient revealed a still adequate learning curve (Table 2, C) and an unremarkable assessment for immediate recall, his performance rate dropped meaningfully for the delayed recalling after the presentation of an interference word list, thus indicating a prominently impaired consolidation of verbal learning matter. In contrast, the patient was able to draw a complex figure in the Rey Complex Figure Test without problems in the immediate and delayed recall. In summary, the patient’s test results were in particular without impairment for logic abstract cognitive functions as well as planning and implementation of actions. However, the results of the WMS and VLMT provide hints for cognitive impairment with regard to the verbal memory. In a structured interview using the ADCS-ADL scale, the patient’s spouse reported declining performance in every-day life activities (achieved score = 39/52 points) during the last 2 years.

**Table 2:** Neuropsychological test results in the CERAD test battery

A				
	Raw	Max	z-score	Evaluation
Verbal Fluency (animals)	18	-	-0,8	Non-significant
Boston Naming Test	15	15	0,9	Non-significant
Mini-Mental Status	28	30	-1,1	Lower border of normal range
Wordlist test	18	30	-0,7	Non-significant
a) Wordlist recall 1st trial	5	10	-0,2	Non-significant
b) Wordlist recall 2nd trial	6	10	-0,7	Non-significant
c) Wordlist recall 3rd trial	7	10	-0,8	Non-significant
Wordlist recall	6	10	-0,5	Non-significant
Intrusions	0	-	0,7	Non-significant
Wordlist Savngs (%)	86%	-	-0,1	Non-significant
Discriminability (%)	90%	100%	-1,5	Mild impairment
Constructional Praxis Test	11	11	0,7	Non-significant
Constructional Praxis recall	8	11	-1,2	Lower border of normal range
Figures-Savngs (%)	73%	-	-1,1	Lower border of normal range
Phonematic Fluency (S-Words)	8	-	-1,1	Lower border of normal range
Trail Making Test, Part A	42	180	-0,1	Non-significant
Trail Making Test, Part B	72	300	1,1	Non-significant
Trail Making Test, B/A	1,7	-	1,1	Non-significant
B				
	Raw	Max	SD	Evaluation
WMS Logical Memory Immediate recall	21	50		avarage
WMS Logical Memory Delayed recall	15	50	<-1	Below average
C				
	Raw	Percentile	T-score	Evaluation
VLMT Consecutiva learning	38	20	41	Lower border of normal range
VLMT Interference	3	<5		Below avarage
VLMT Recall	5	<5	29	Below avarage
D				
	Performance	Evaluation		
Clock-Drawing Test	Mild vsuo-constructive deficits score=1-2	average		
Rey Complex Figure Test	Normal range	average		

### Structural MRI results

As shown by two previous MRI scans, the structural MRI revealed an extended arachnoid cyst in the left fronto-temporal region, with a midline shift about 12 mm to the right (Fig. 1). A compression of the right lateral ventricle, a displacement of the mesencephalon to the right as well as a displacement of the arteria cerebri media in the M1- and M2 segments were shown. On the right cerebral hemisphere a slight global brain volume reduction was detected. The right temporal horn was slightly enlarged (Scheltens score 2) as sign of medio-temporal lobe atrophy typical for Alzheimer’s disease [15]. There was no relevant microangiopathy (Fazekas score 0).

### Functional imaging

**Fig. 2:**
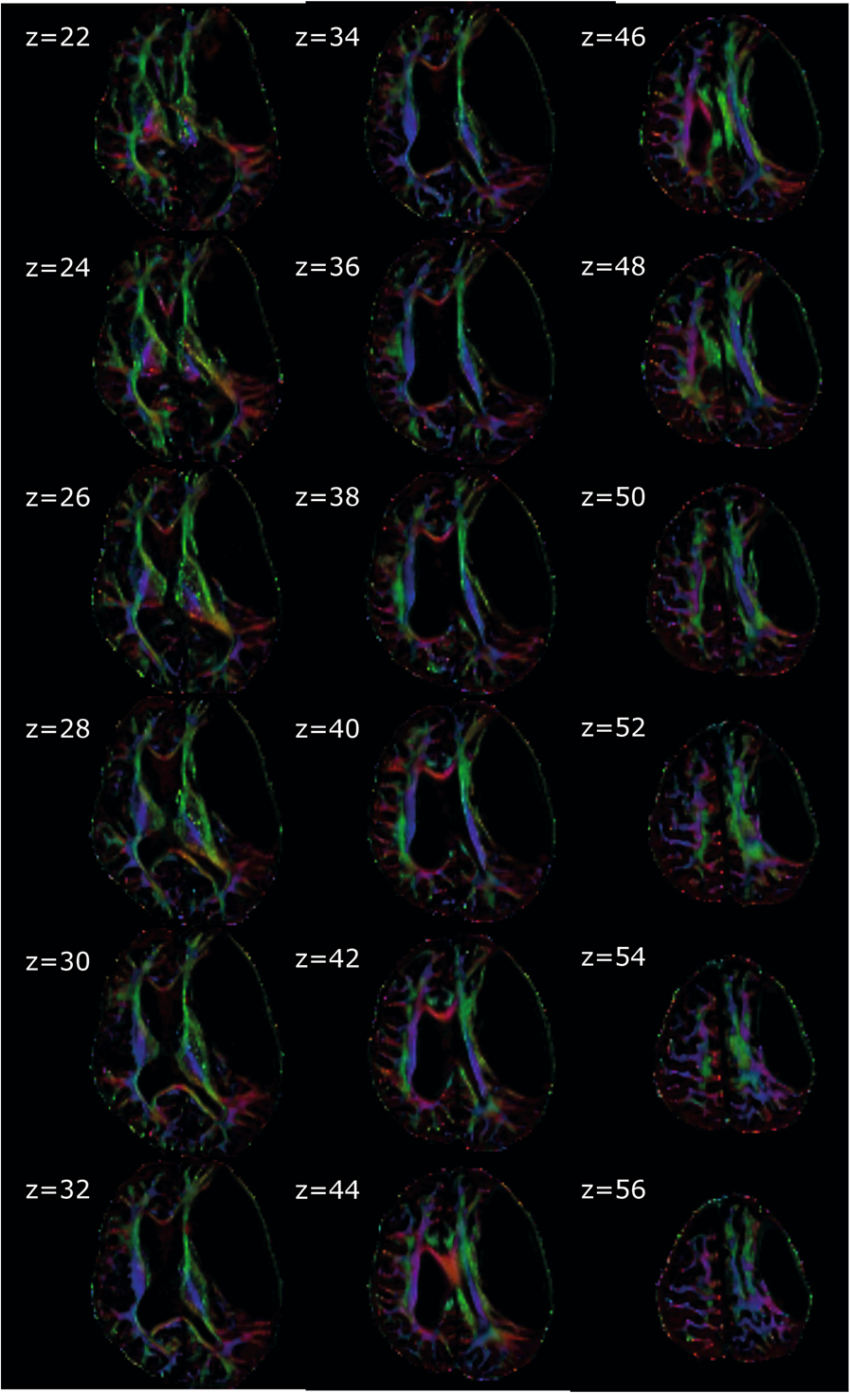
Images of first eigenvector overlaid on fractional anisotropy (FA) images. The brightness of the vector colors in each voxel is modulated according to the values in the corresponding FA image, indicating the axis of diffusion. The colors red, green and blue correspond to the directions left/right, anterior/posterior and inferior/superior, respectively. The slice position in axial direction is given by its z coordinate next to the image

Since the structural MRI had revealed a marked mass effect on the left hemisphere (Fig. 1) induced by the giant cyst, we next aimed at exploring impairment of functional integrity in particular to the left hemisphere due to the arachnoid cyst. Diffusion tensor imaging (DTI, Fig. 2) provided a first hint of relative well-preserved structures of fiber bundles in the left hemisphere compared to the right one.

**Fig. 3:**
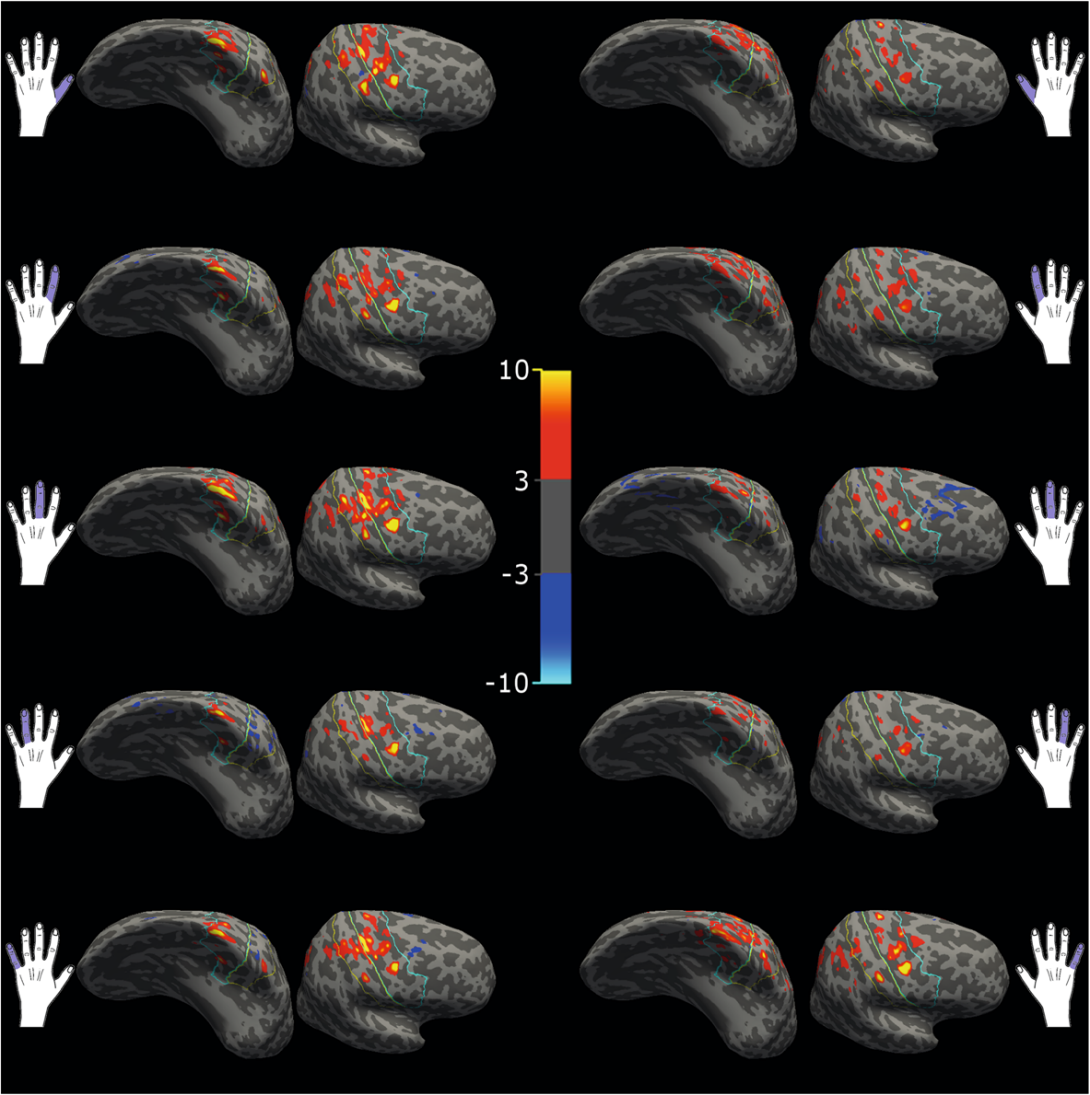
Functional magnetic resonance imaging results overlaid on the inflated left and right hemisphere reconstruction. The rows 1 to 5 depict activation patterns in response to digit 1 (first row) to digit 5 (last row). The first and second columns depict activation in the left (first column) and right hemisphere (second column) in response to left hand stimulation. The third and fourth columns depict activation in the left (third column) and right hemisphere (fourth column) in response to right hand stimulation. Activation is shown as ‘significances’, ie, −log10(p). So, for *p* = .001, sig = 3. Colour code ranges from 3 to 10 as illustrated by the color bar in the center. Additionally outlines of pre- and postcentral gyrus are depicted in blue and yellow, corresponding to parcellation results depicted in Additional file 1: Figure S1

Several studies have suggested a direct interplay between cognitive and motor functioning in the healthy elderly [16–18]. We thus performed fMRI with a sensorimotor task (visually cued movements of individual digits in the scanner, see methods) to examine functional integrity of the left hemisphere despite the giant cyst. Preserved motor representation was shown by co-localization of the motor representation of the fingers and parcellation results for primary motor and sensory cortex in the left and right hemisphere (Fig. 3), including an intact representation of the right fingers in tissue which was roughly localized at the position of the primary motor and sensory cortex (Additional file 1: Figure S1, Additional file 3: Figure S3). Although the cyst had a huge mass effect squeezing and smashing the left hemisphere towards the right one mapping of the functional areas was localized similarly in both hemispheres. Left hand movements were better represented in both hemispheres compared to the right hand, reflecting the predominant left-handedness of our patient. The bilateral presentation of both hands (although weaker in the right hand) may be explained by the relearning of right hand-usage for writing at school (Table 1). In summary, our results suggest the structural and functional preservation of the left hemisphere independently of the arachnoid cyst.

### Amyloid-PET result

**Fig. 4:**
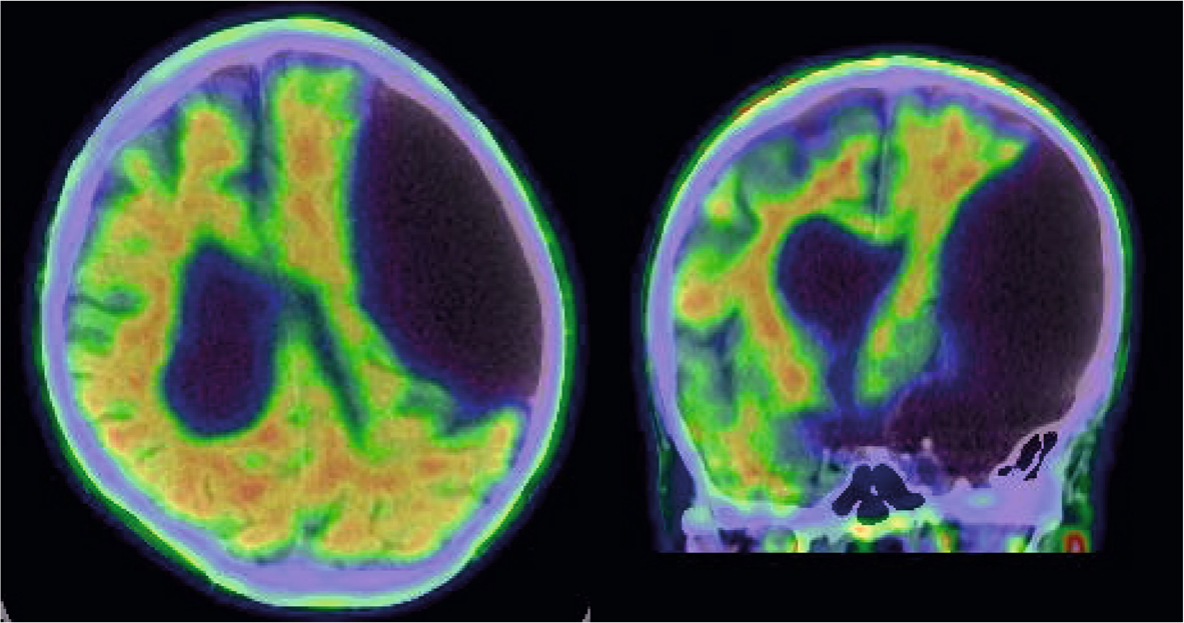
Coronal and horizontal brain slices of Amyloid-PET imaging. F18-FBB (Florbetaben) tracer was used depicting amyloid-beta burden in both grey and white matter. The border between grey and white matter is indistinguishable thus indicating a pathological cortical amyloid-beta deposit

The results of the functional imaging made tissue damage and thus a consecutive cognitive decline due to the giant arachnoid cyst less probably. The structural magnetic resonance imaging however had elucidated a first hint for another underlying neurodegenerative cause for the beginning cognitive impairment: Medio-temporal lobe atrophy in the right hemisphere, typical for Alzheimer’s disease. Because of the unfavorable risk/benefit balance ratio for lumbar puncture in this patient we decided for an amyloid-PET for investigation of Alzheimer pathophysiology. Pathologically increased cortical Amyloid deposition was detected in both white and grey matter (Fig. 4), typical for Alzheimer pathophysiology.

We also tested for an apolipoprotein E4 allele. However the patient was negative for an APoE-Genotype suggestive for an advanced manifestation of Alzheimer dementia.

The patient is now followed up in the memory clinic for further cognitive decline or progression into Alzheimer’s dementia.

### Review of literature

Neuroimaging has revealed a large left frontotemporal cerebrospinal fluid-filled cyst in our patient. Although arachnoid cysts may develop anywhere in the central nervous system, most cysts have been reported in the middle cranial fossa [19]. We have performed a systematic search of literature to enlighten the typical location and clinical manifestation of intracranial cysts presented in literature and potential interaction with the manifestation of dementia. We found 14 clinical studies and case reports (Table 3 [2, 6, 7, 20–29]), which presented subjects with frontotemporal arachnoid cysts and cognitive decline or other neurological symptoms. We analyzed a total of 47 patients including 28 men (59.6%) and 19 women (40.4%). Average age was 50.9 years in men and 47.9 years in women. Comparable to our patient, the location of the arachnoid cysts was more pronounced in the left hemisphere (68.1%), versus the right hemisphere (21.3%) versus an interhemispheric location (10.6%). Our data are in accordance with others claiming that cysts are commonly located on the left side with males most commonly having cysts in this area [25].

**Table 3:** Overview of the here reviewed 15 clinical studies and case reports, which presented subjects with frontotemporal arachnoid cysts and cognitive decline as well as other neurological symptoms

Piece of literature	# of patients	Age of patients	Sex	Hemisphere	Interhemispheric	Cognitive impairment	Other symptoms	Surgery
Amin, 2013	1	65	m	right	no	yes	spastic left sided hemiparesis, late onset seizures	no
Bohnen, 2016	1	70	m	left	no	yes	gait disturbance	no
Gjerde, 2013	22	22-68	12x m/ 10xf	14x left, 8x right	no	yes (4 pat.)	vertigo (2 pat.), headache (19 pat.), epilepsy (2 pat.)	yes
Golaz, 1993	1		f		yes	yes	no	no
Hishikawa, 2002	1	64	f	left	no	yes	headache, speech disturbance	yes
Hishikawa, 2002	1	73	f		yes	no	right hemiparesis	yes
Kotil, 2007	1	70	f		yes	yes	vertigo, behavioral disturbance	yes
Lebowitz, 2006	1	33	m	left	no	yes	no	no
Miyaji, 2012	1	68	m	left	no	yes	headache, acalculia, agraphia, finger agnosia	no
Ramtahal, 2006	1	61	f		yes	yes	gait disturbance, incontinence	yes
Shirakawa, 1991	1	72	f	right	no	yes	gait disturbance, incontinence	yes
Sugimoto, 2016	1	72	m		yes	yes	gait disturbance, dizziness	yes
Wester, 1995	13		11x m/ 2x f	left	no	no	seizures (3 pat.), hemiparesis (2 pat.),	yes
Zwagerman, 2016	1	49	f	left	no	no	staring spells, expressive dysphasia, tremor	yes

In the 47 patients analyzed here (Table 3), main predilection site was the temporal lobe (70.2%), with the frontal lobe second (8.5%) or in both lobes (4.3%) versus other locations (17.0%). 42.8% of all subjects described presented with cognitive impairment which ranged from word finding difficulties to signs of dementia. In contrast to our patient, headache was the most prominent symptom in 72.3% of patients. Other typical symptoms associated with arachnoid cysts were epilepsy and gait disturbance/vertigo (both in 14.9% of patients) as well as hemiparesis, incontinence and speech impairment (in 8.5% of the patients analyzed). However, most literature on cysts and cognitive decline is published by neuro-surgical departments. 89.3% of the patients included in the studies reviewed here received surgical treatment in form of cysto-peritoneal-shunt surgery or cystectomy, thus indicating a reporting bias of mostly publishing cases with symptomatic cysts requiring surgical intervention. Long-term studies examining arachnoid cysts and assessing cognitive function over time are missing so far.

## Discussion and conclusions

Our patient revealed two potential etiological factors for the development of cognitive decline: The giant arachnoid cyst reducing his neuronal plasticity and Alzheimer’s disease [30]. As shown in our case report an extensive work-up was required to dissect the contribution of each disease entity although their interaction remains a matter of debate. Our case particularly illustrates that a giant arachnoid cyst may leave the brain tissue intact but might mask an underlying neurodegenerative disorder such as Alzheimer’s dementia. It also illustrates the diagnostic value of biomarkers, e.g. medio-temporal lobe atrophy (Scheltens Score 2) and increased Amyloid-PET imaging. Thus, the diagnosis of prodromal Alzheimer dementia (AD) / mild cognitive impairment due to AD was established in this patient [31].

Although elevated tracer binding can also be found in normal older volunteers without cognitive deficits, with the proportion of “positive cases” ranging from 10 to 30% depending on the age of the cohort and the threshold for defining the pathological tracer enhancement [32–35], the preponderance of current literature proposes that enhanced amyloid-beta signal in non-demented subjects is associated with decreased episodic memory performance and structural/functional brain changes suggestive of incipient Alzheimer’s disease [36]: In a retrospective study [37], amyloid-beta positive PET scans were more common in subjects with declining cognitive test scores (70%) than in those with stable scores (17%).

Our findings in the cognitive assessment are in line with previous studies showing a rather heterogeneous picture of cognitive deficits across different domains, lacking clear specificity of cognitive and neuropsychological changes following prodromal AD/MCI due to AD. The often expansive lesions due to arachnoid cysts may cause a reorganization of cortical functions. This reorganization can vary to a large extent between patients, which is reflected in changes in a variety of cognitive functions. According to a review article [38], these functions encompass verbal perception and memory, complex verbal tasks, visuospatial functions and visual attention and dichotic perception and memory [7] of which not all have to be impaired in every patient. The interpretation of cognitive impairments thus provide some evidence on the specificity of the reorganization and compensation, yet, this needs to be treated with caution as various factors co-determine such effects.

Furthermore, the application of different imaging modalities and biomarkers helped the neurosurgeons to triage our patient: the patient was followed-up by the neurosurgeons several times for a surgical intervention as clinically a mild cognitive impairment became apparent. However, when Alzheimer’s disease was revealed as the leading underlying cause of cognitive decline rather than tissue damage from the arachnoid cyst, the neurosurgeons decided against a cystectomy. As there was no cyst size progression and no further neurological symptoms a benefit from the surgery was highly questionable for the patient.

Moreover, this case study is also an impressive demonstration of neuronal plasticity fully compensating for even large intracranial pathologies. Most intracranial arachnoid cysts remain clinically silent [1], reflecting the tremendous plastic capacity of the central nervous system to compensate for even large structural abnormalities. As arachnoid cysts are usually congenital, the brain fully adapts to the special circumstances during development, a condition which could be also found in our patient: The 66-year old lived his life rather unaffected by the giant arachnoid cyst, he would not be aware of until the age of 63. The patient remembers of wearing headdresses of adults at a very young age and being teased because of his head size as a child suggesting the congenital nature of the arachnoid cyst. Similar phenomena have been described in children with intractable seizures who underwent surgery with resections of entire brain regions or callosotomy [39, 40].

The application of different neuroimaging modalities revealed structural and functional perseveration of the left hemisphere despite being displaced and squeezed by the giant cyst: Representation of finger movements was sustained at a rather similar position in the left hemisphere compared to the right sensorimotor cortex. Interestingly, bilateral representation in particular of the left hand was revealed and even pronounced in the right hemisphere, which might be explained by the ambidexterity of the patient, being originally left-handed but trained right-handed. We chose a sensorimotor task for the fMRI experiment to map in particular the left hemisphere, as the giant arachnoid cyst had a strong mass effect on this brain region. The goal of the fMRI was to detect any functional map shift and loss of functional integrity (together with structural alterations found in the DTI (Fig. 2)), which could also impact cognitive functions. Since we did not aim at detecting the neuronal correlate of the cognitive decline but at understanding the structural and functional integrity despite the giant cyst, a cognitive task with fMRI would have been less helpful. Although we cannot rule out that memory function is still impaired despite of the intact motor function, normal sensorimotor integration may be also correlative for cognitive performance as discussed by others [16, 18] and vice versa: Abnormal sensorimotor integration correlates with the cognitive profile in neurological diseases [41].

However, it remains a matter of debate, if the plastic compensational mechanisms of the brain decline over time, and thus expose patients with even clinically silent brain pathologies to cognitive impairment. Although a connection between arachnoid cysts and cognitive sequelae has been proposed for over 20 years [42], the impact of clinically silent cysts on cognitive decline remains largely unknown due to the subtlety of dysfunction. Rabiei et al. [43] found that subjects with and without cysts had the same frequency of headache, dizziness, previous head trauma, cognitive impairment and depressive symptoms without an increased prevalence of dementia, depression, epilepsy or previous hip fracture. Nonetheless a relationship between the onset of Alzheimer’s pathology and the arachnoid cyst might be plausible: As previously postulated by Silverberg et al., 2003 [44] we speculate that the existence of the arachnoid cyst may modify intracranial pressure ratios, leading to reduced perfusion and metabolism in the surrounding cortical regions. Furthermore, the cyst might perturb CSF circulatory pathology which might result – along with genetic factors such as apolipoprotein E4 Aβ -in the accumulation of amyloid-β peptide, microtubular associated protein τ (MAP τ) and other toxins. An increase in the steady state concentration of Aβ influences Aβ aggregation which is causally und pathognomonic in Alzheimer’s disease [45, 46]. In particular soluble Aβ is potentially neurotoxic affecting normal neuronal function and even causing cell death [47, 48]. Since our patient presented with apolipoprotein E ε3/3 wildtype allele, this genotype will not have contributed to the early manifestation of Alzheimer dementia. Thus it may be speculated that the impaired CSF flow dynamics due to the giant cyst might have caused reduced amyloid clearance inducing Alzheimer pathology. Longitudinal studies in patients with huge arachnoid cysts might become important to detect symptoms of cognitive decline early and thereby open a window of opportunity for early therapeutic intervention to prevent or slow down further cognitive deterioration [49].

One major lesson to be learned from the case illustrated here is, that patients presenting with large intracranial brain cysts and cognitive decline require a closer look and pure clinical examination and structural MRI might be insufficient to triage patients either to neurosurgery or watchful waiting. The review and study presented here may thus initiate further discussion for a modus operandi for patients with large intracranial cysts: We suggest [1] a detailed neurological and neuropsychological examination as well as MRI of the brain [2]. The repetition of all procedures is crucial to detect the development of cognitive decline and neurological symptoms over time. The cyst may also become symptomatic due to growth progression and may thus require surgical intervention [3]. An additional neurodegenerative component should be evaluated either by biomarkers in the cerebrospinal fluid or amyloid-PET imaging [4]. Additional fMRI may become a useful assessment to study functional integrity and for risk analysis of cognitive deterioration: Patients with less prominent functional map changes and no neurodegenerative comorbidity may be at lower risk for a cognitive decline. In contrast, large functional map shifts may be associated with cognitive impairment due to a decline of plastic compensation in aging.

The case presented here illustrates that in patients with large intracranial brain cysts and cognitive impairment pure clinical examination and structural MRI might be insufficient to triage patients. Our case report suggests the diagnostic value of rigorous neuroimaging modalities including DTI, fMRI and amyloid-PET imaging as well as biomarkers in the cerebrospinal fluid to distinguish tissue damage induced by large cysts versus a neurodegenerative pathology as underlying cause of cognitive decline.

## Additional files

Additional file 1: **Figure S1.** Images of parcellation results for precentral and postcentral gyrus overlaid on T1 weighted structural image. The colors blue and yellow correspond to the pre- and postcentral gyrus. The slice position in axial direction is given by its z coordinate next to the image. (PDF 10040 kb)

Additional file 2: **Figure S2.** Images of segmentation results. The colors red, white and purple correspond to the grey matter, white matter and cerebrospinal fluid. Additionally parcellation results of precentral (cyan) and postcentral (yellow) gyrus are depicted. Slice position is given by x, y and z coordinates. (PDF 221 kb)

Additional file 3: **Figure S3.** Images of atlas mapping in volume space and surface space. The colors correspond to the different areas as assigned by atlas mapping. The top left shows respective results in surface space of the left hemisphere, the top right shows respective results in surface space of the right hemisphere. The bottom shows an exemplary axial view of atlas mapping in volume space at z = 70. Red and blue areas depict precentral (blue) and postcentral (red) gyrus. (PDF 2914 kb)

## Abbreviations

AD: Alzheimer Dementia
ADL: Activity of Daily Living
A-PET: Amyloid-Positron Imaging Tomography
BW: Bandwidth
CERAD: Consortium to Establish a Registry for Alzheimer’s Disease
DTI: Diffusion tensor imaging;
FoV: Field of view
MCI: Mild cognitive impairment
MDDW: Multi-directional diffusion weighting
MMSE: Mini-Mental State Examination
SE: Spin echo
TE: Echo time
TR: Repetition time
TSE: Turbo spin echo sequence
VLMT: Verbal Learning and Memory Test
WMS-LM: Wechsler Memory Scale logical memory
