# Supplementary figures and images for: Case report: a giant arachnoid cyst masking Alzheimer’s disease
Source: BMC Psychiatry. 2019 Sep 5;19:274. doi: 10.1186/s12888-019-2247-8 (PMC6728996; doi:10.1186/s12888-019-2247-8)

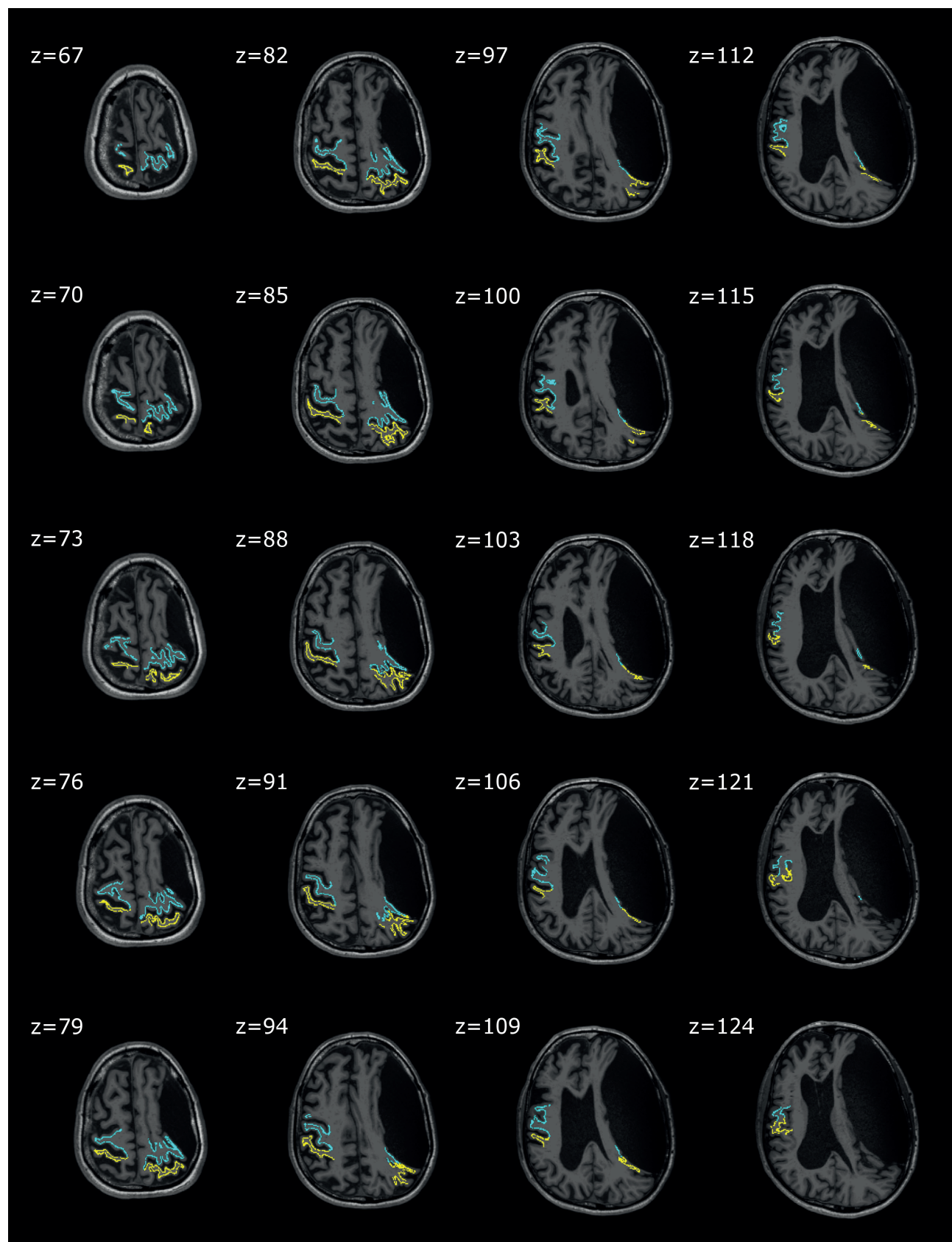

Supplement: Supplementary file 1 — Figure S1. Images of parcellation results for precentral and postcentral gyrus overlaid on T1 weighted structural image. The colors blue and yellow correspond to the pre- and postcentral gyrus. The slice position in axial direction is given by its z coordinate next to the image. (PDF 10040 kb) [file 12888_2019_2247_MOESM1_ESM.pdf]

x=146

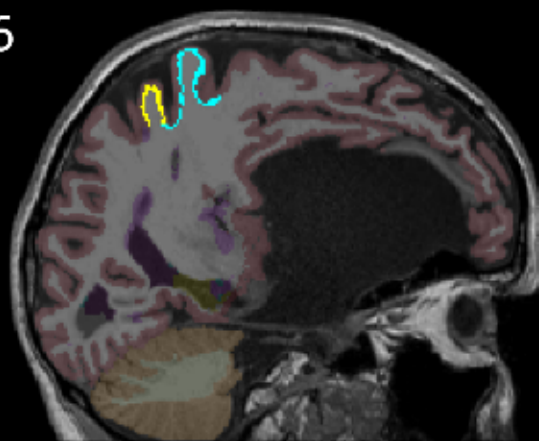

y=98

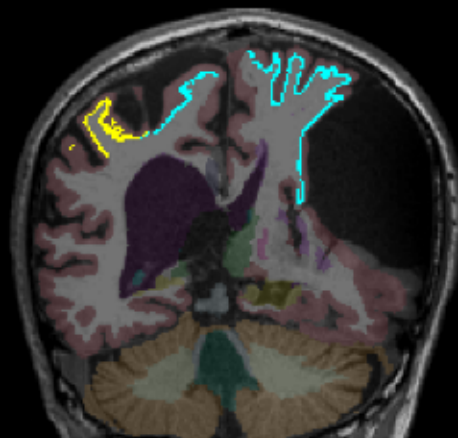

z=86

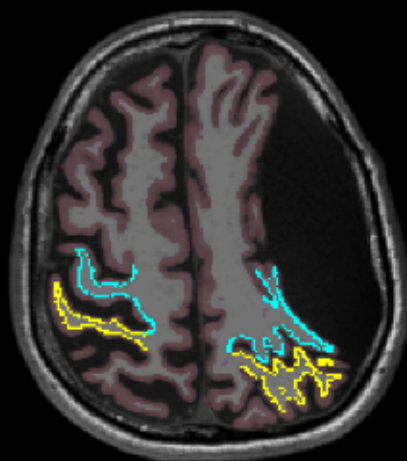

Supplement: Supplementary file 2 — Figure S2. Images of segmentation results. The colors red, white and purple correspond to the grey matter, white matter and cerebrospinal fluid. Additionally parcellation results of precentral (cyan) and postcentral (yellow) gyrus are depicted. Slice position is given by x, y and z coordinates. (PDF 221 kb) [file 12888_2019_2247_MOESM2_ESM.pdf]

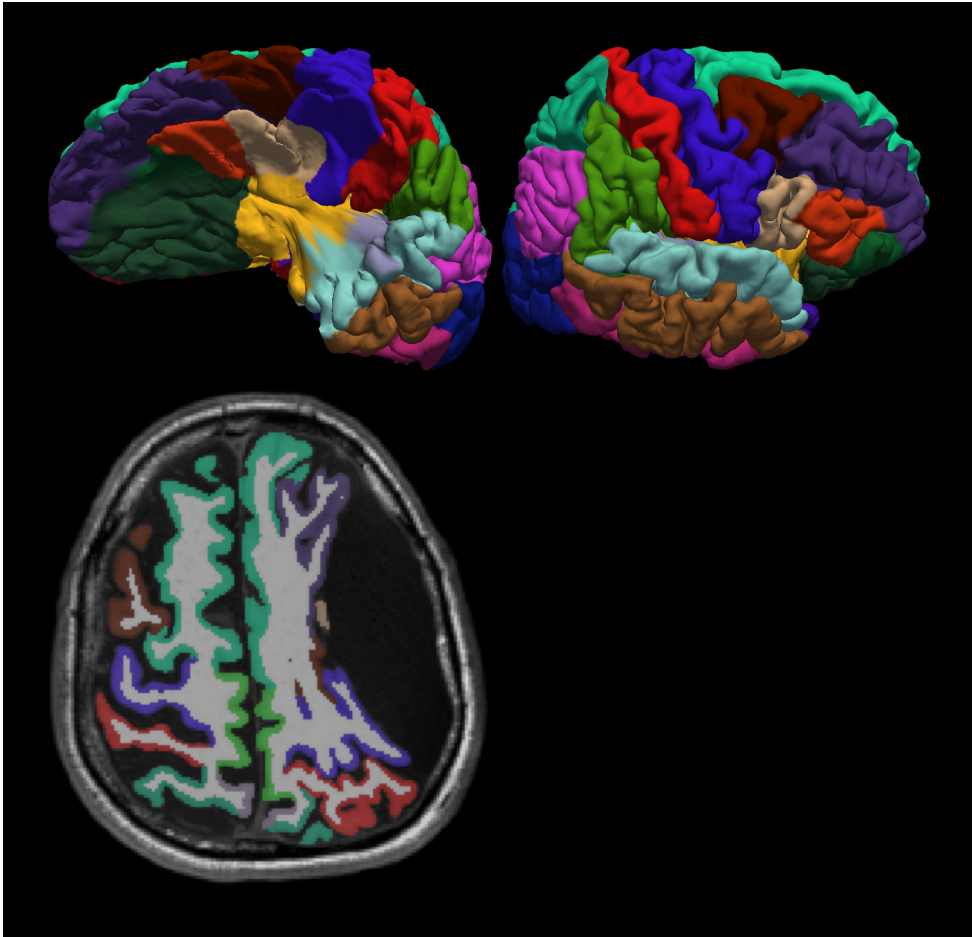

Supplement: Supplementary file 3 — Figure S3. Images of atlas mapping in volume space and surface space. The colors correspond to the different areas as assigned by atlas mapping. The top left shows respective results in surface space of the left hemisphere, the top right shows respective results in surface space of the right hemisphere. The bottom shows an exemplary axial view of atlas mapping in volume space at z = 70. Red and blue areas depict precentral (blue) and postcentral (red) gyrus. (PDF 2914 kb) [file 12888_2019_2247_MOESM3_ESM.pdf]
